# Duodenal diverticulitis: a difficult clinical problem

**DOI:** 10.11604/pamj.2017.27.286.13509

**Published:** 2017-08-23

**Authors:** Houcine Maghrebi, Zoubeir Bensafta

**Affiliations:** 1 Surgery Department A-Rabta Hospital Tunis, Tunisia

**Keywords:** Duodenal diverticulum, surgery, radiology

## Image in medicine

A duodenal diverticulum is a pouch attached to the duodenum which may be present in 20% of the population. Although they are common entities, symptoms caused by duodenal diverticula are relatively rare and complications such as diverticulitis remain a difficult clinical problem. Nonoperative management has emerged as a safe, practical alternative to surgery in selected patient. We present a rare case of duodenal diverticulitis and its successful conservative management. A 57-year-old man was admitted to the Emergency Department with a 4-day history of epigastric and right upper quadrant pain. The patient claimed a six month history of abdominal pain and weight loss. Physical examination shows fever and tenderness of the epigastric and right upper quadrant. Laboratory tests revealed an elevated leukocyte count with normal liver tests, lipase level. Abdominal X-ray showed no intra-peritoneal free air. Computer tomography of the abdomen reveals an infected duodenal diverticulum with infiltration of neighboring fat. The patient was admitted to the acute care surgical service for a conservative management: nasogastric suction, bowel rest, intravenous antibiotic therapy, parenteral nutrition with a close clinical observation. The patient improved and was discharged on hospital day 10 without complications.

**Figure 1:**
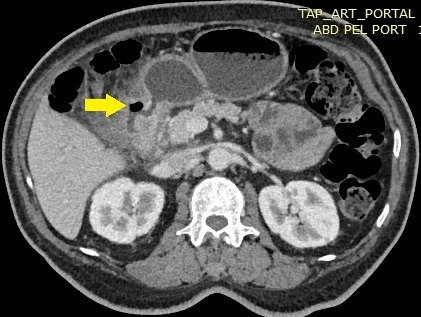
CT scan showing the duodenal diverticulitis

